# Supplementary figures and images for: The piggyBac derived transposase 5 (PGBD5) can interact with human piggyBac-like elements
Source: bioRxiv. 2025 Aug 2:2025.07.31.667870. Preprint. [Version 1] doi: 10.1101/2025.07.31.667870 (PMC12324416; doi:10.1101/2025.07.31.667870)

**A.**

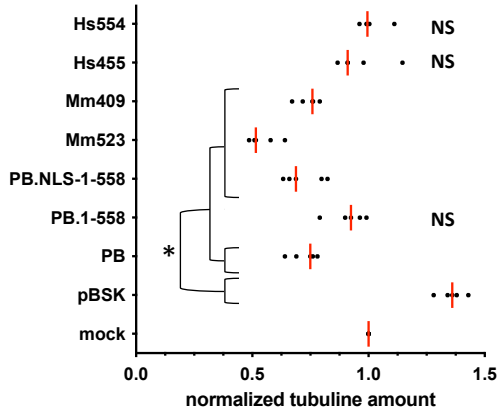

**B.**

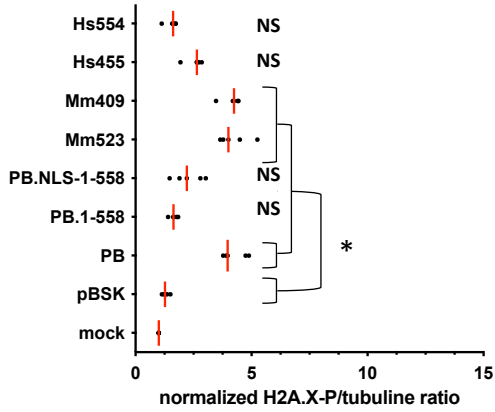

**C.**

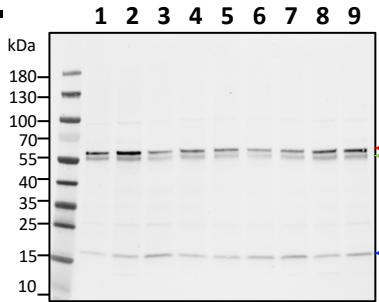

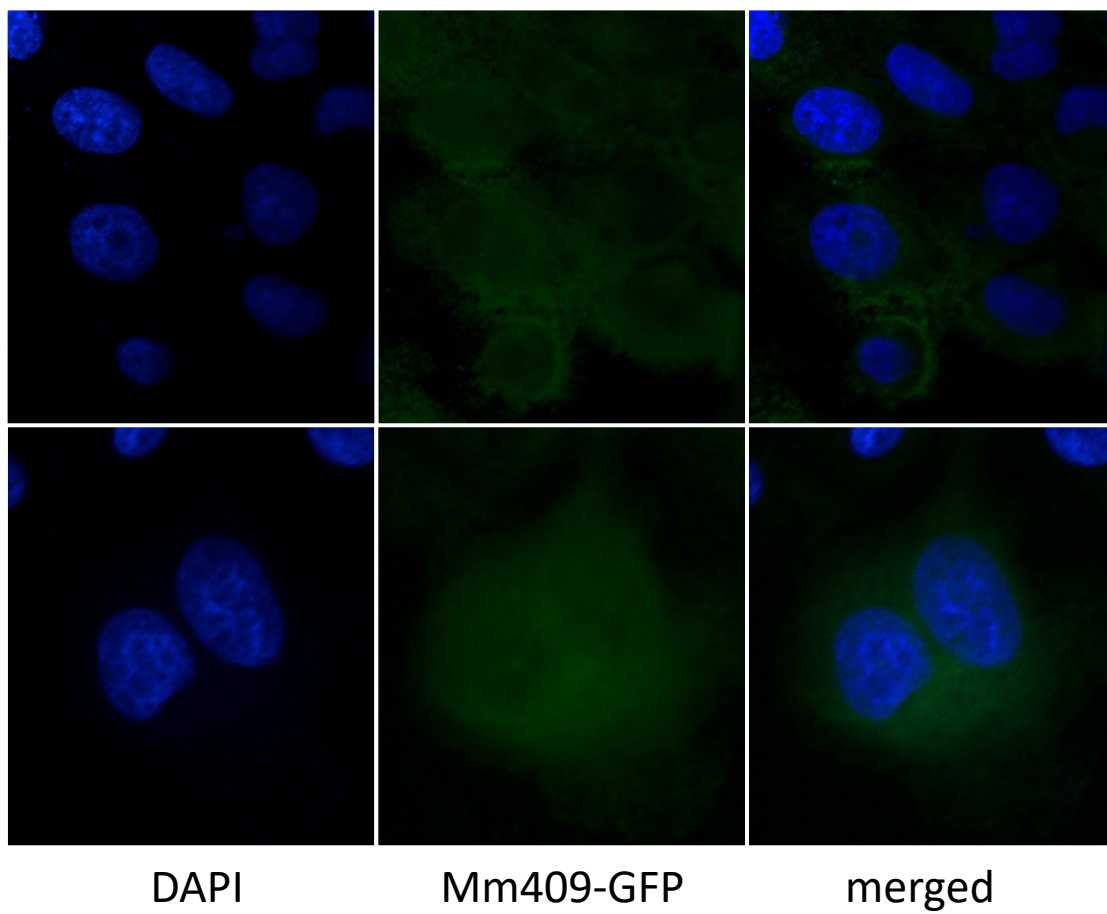

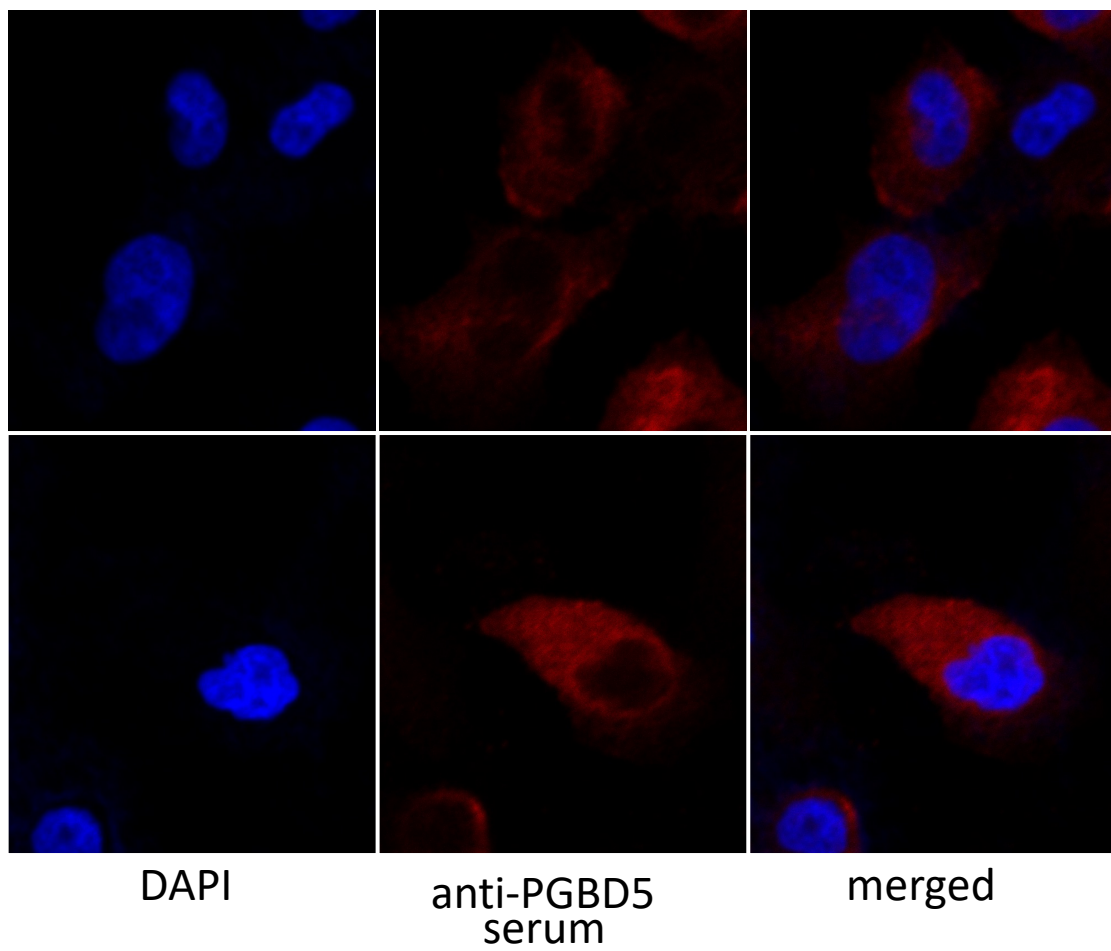

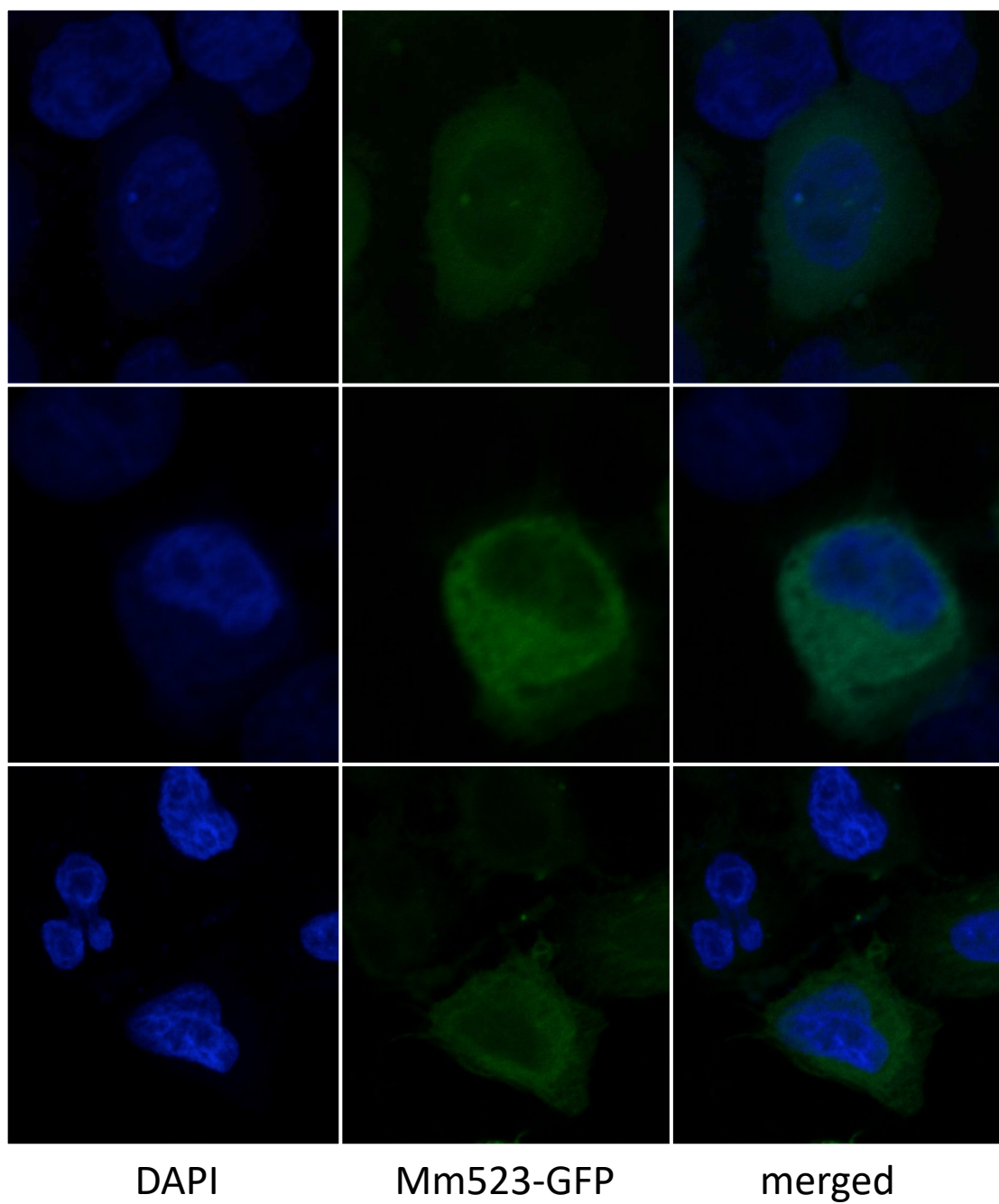

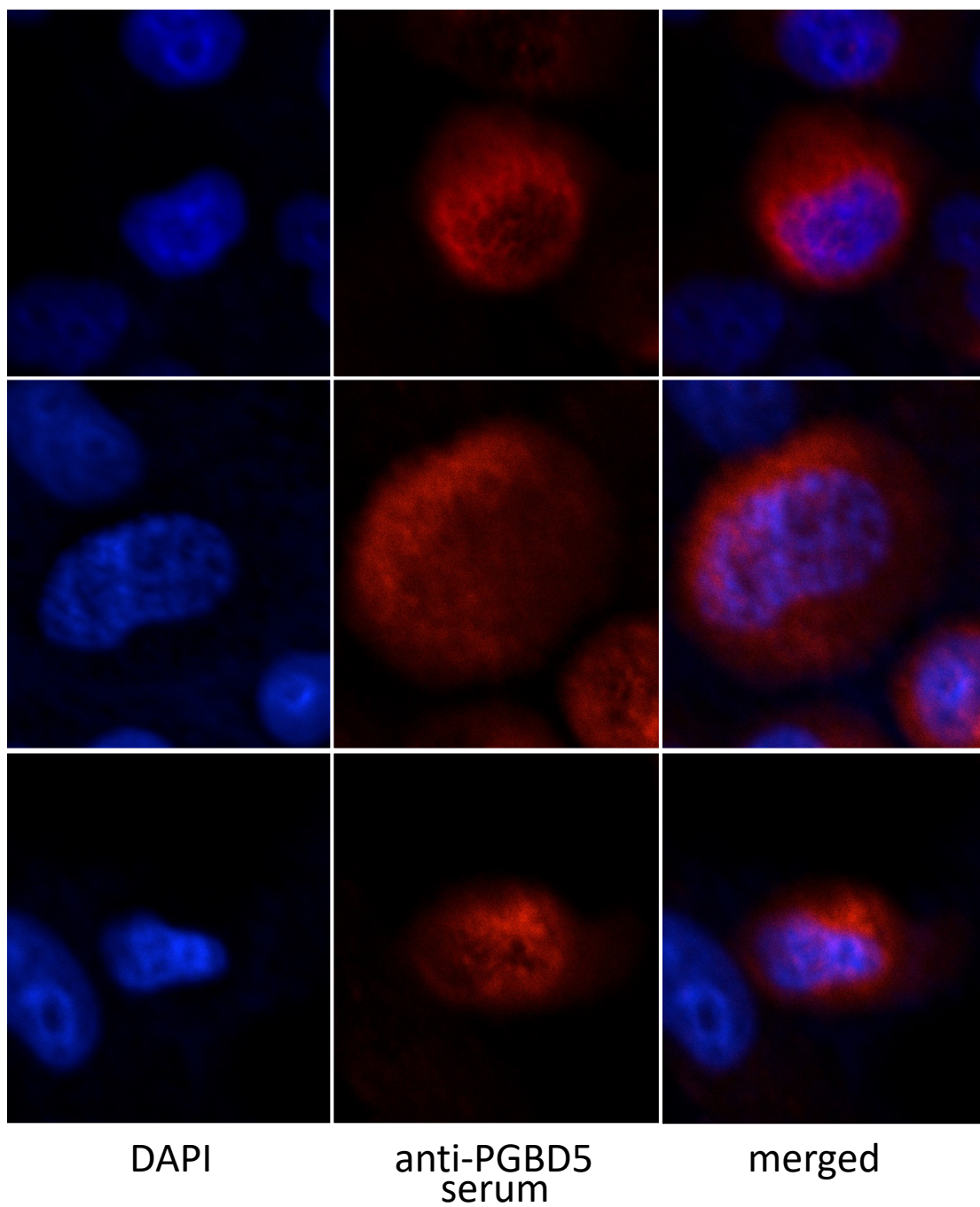

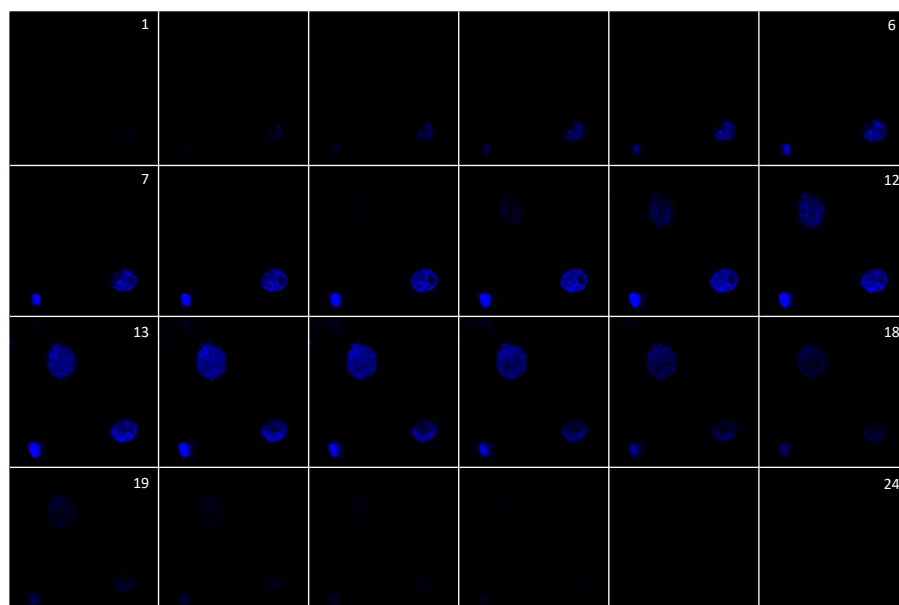

DAPI

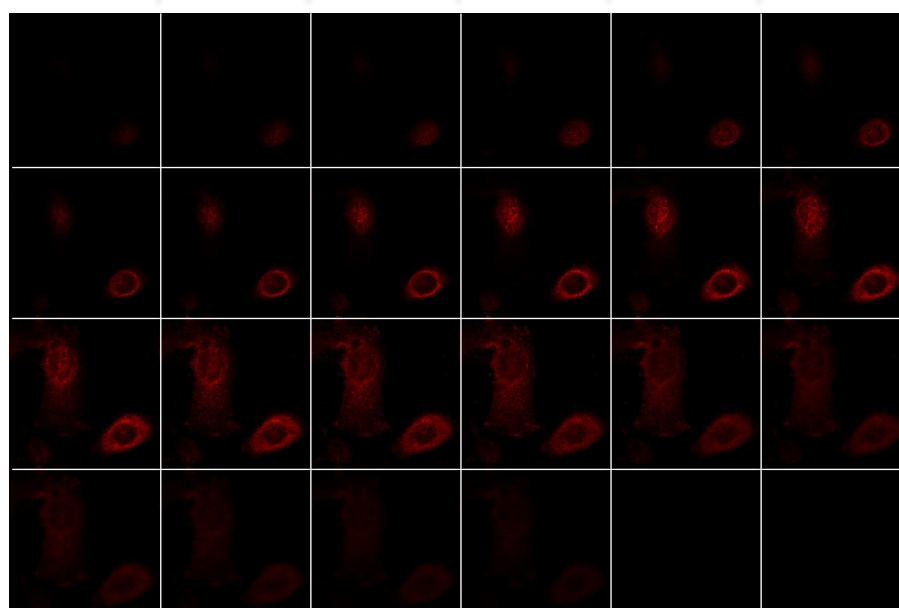

anti-PGBD5  
serum

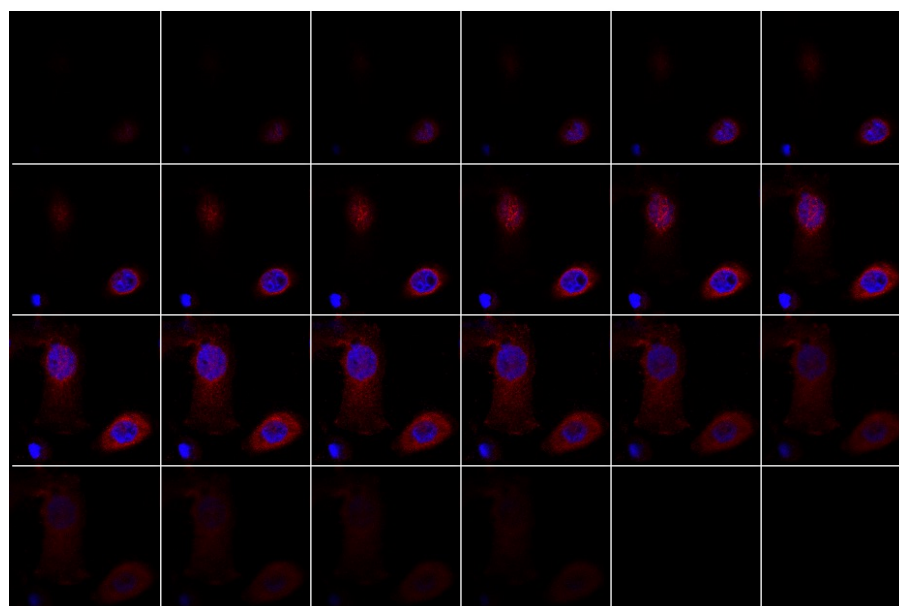

merged

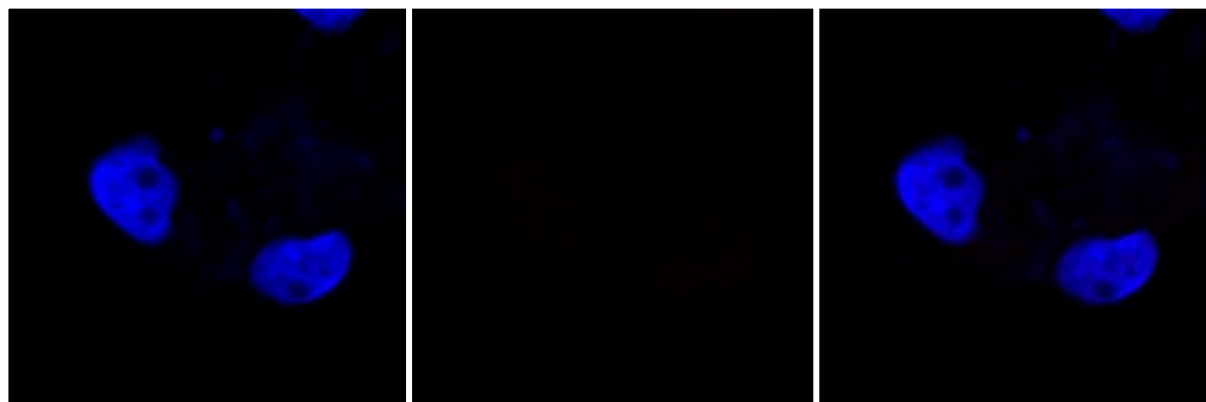

DAPI

Preimmune sera

merged

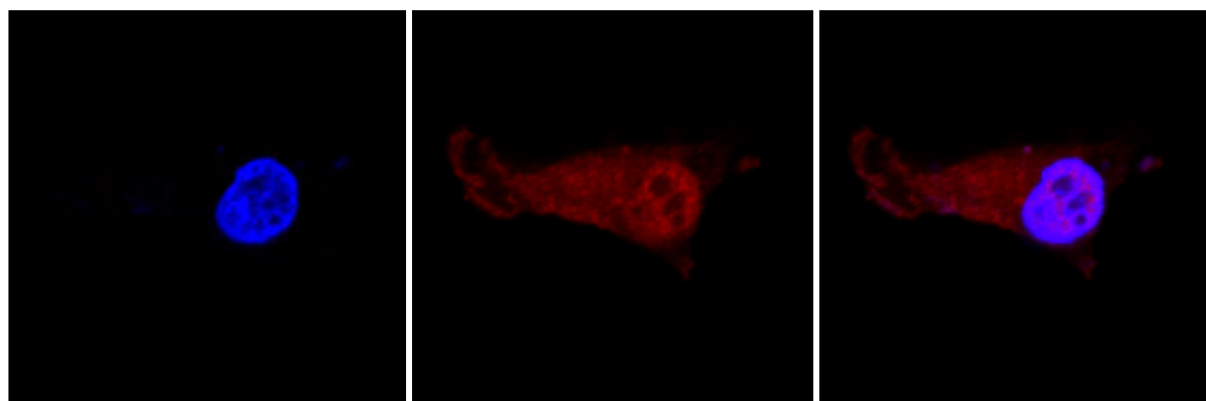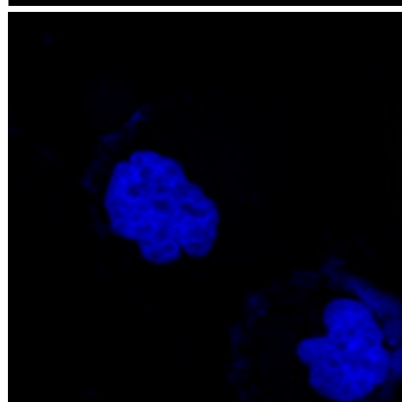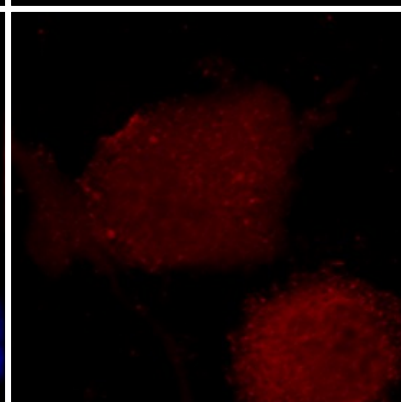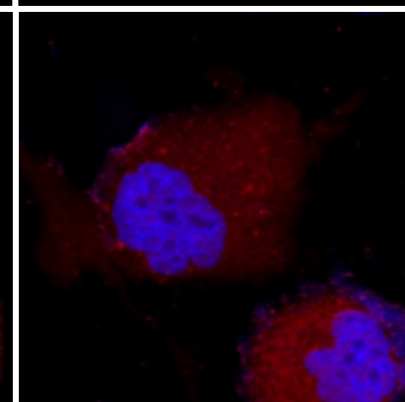

DAPI

anti-PGBD5  
serum

merged

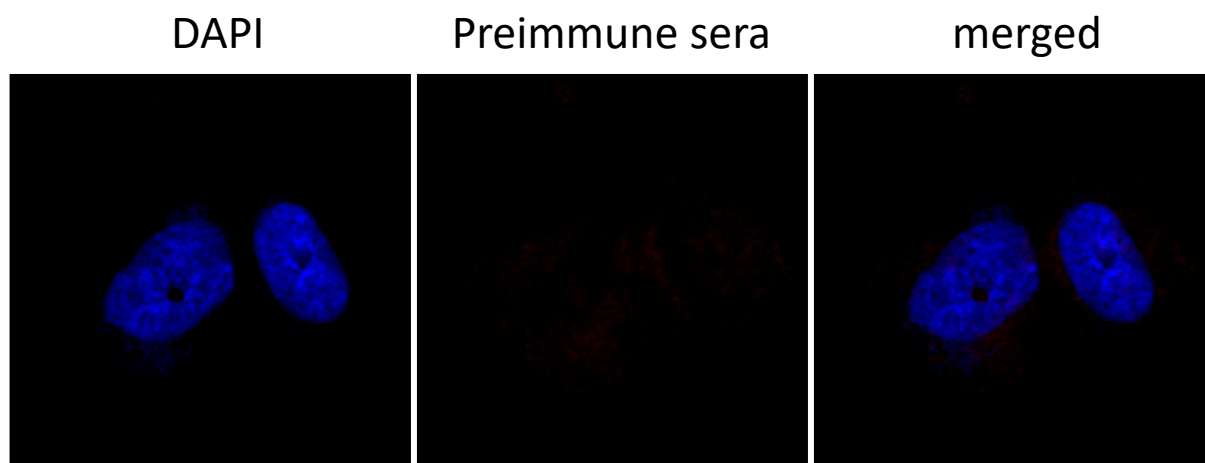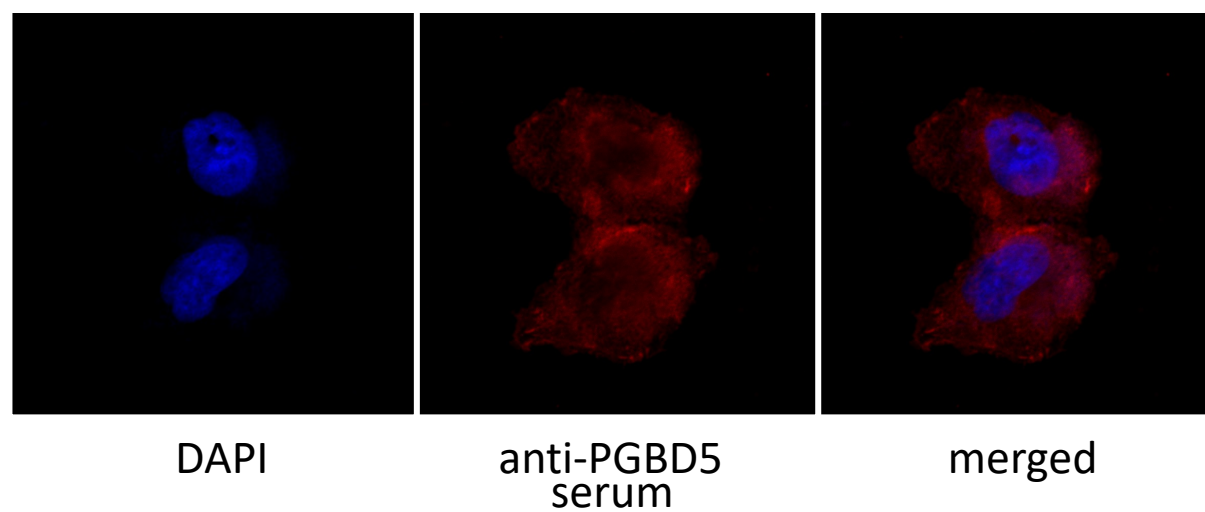

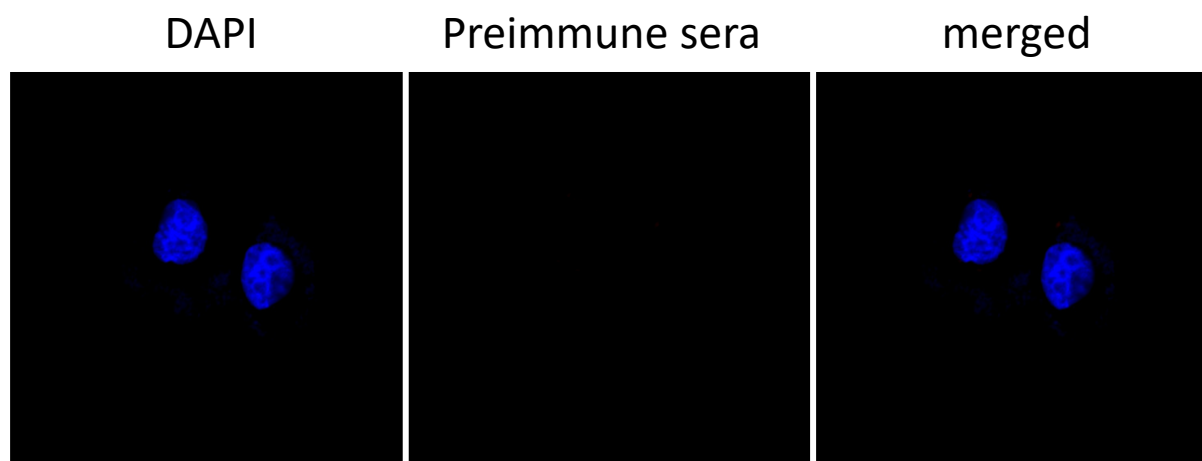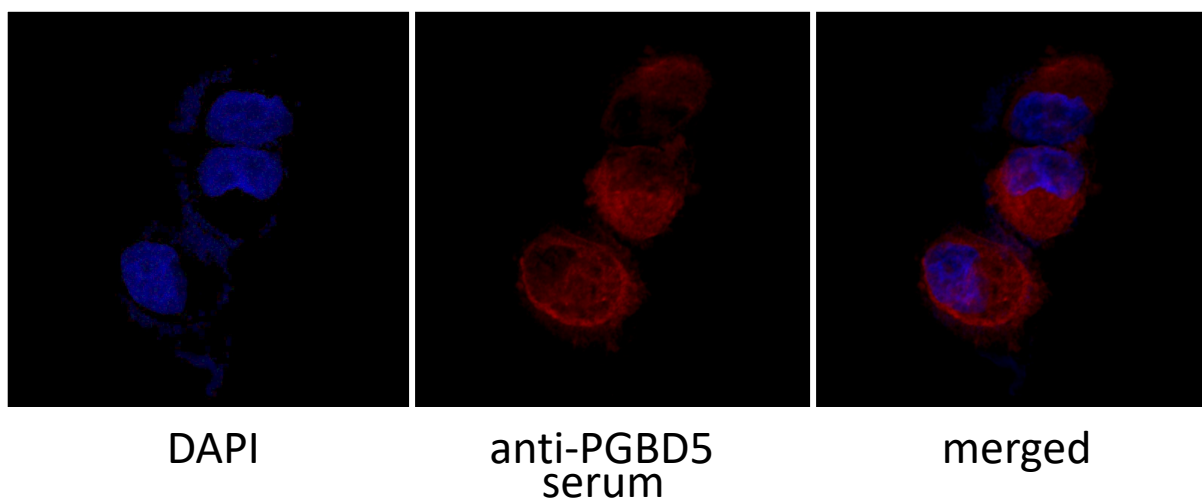

Supplement: Supplement 1 — Figure S1. Impact of PGBD5 isoforms and PB variants on rates of H2A.X phosphorylation and survival in HeLa cells. (A), normalized a-tubulin amount calculated from quantification on western blots made with protein extracts from 105 HeLa cells transfected with 400 ng and a transposase/transposon donor ratio of 1 and, (B), graphic representation of H2A.X-P/α-tubulin. Transfection rate was ~75% and was evaluated in parallel using a pGL3-DsRed-Kan (a gift of Pr Nicolas Mermod, Lausanne, Switzerland). (C) Western blot analyses of phosphorylated γ-H2AX, α-tubulin, and PRPF19 in HeLa cell extracts transfected with each of the four PGBD5 isoforms and the 3 PB variants. Each protein extract was prepared 24 hours post transfection from the complete cellular content of one well of a 24-well plate. In each plot, the red lines represented the median calculated from 5 experiments fully replicated. In A and B, data were normalized on pBSK and in C and D on the mock. * and brackets, and NS indicated a significant and a non-significant Krustal-Wallis test (p>0.05) between results obtained with PB, PB.1–558, PB.NLS-1–558, Mm523, Mm409, Hs455 or Hs554, and pBSK. In (C), for the revelation of phosphorylated γ-H2AX, α-tubulin and PRPF19, mouse anti-phospho-H2A.X and anti a-tubulin antibodies were used while and a rabbit polyclonal anti-PRPF19 was used. Secondary antibodies were a donkey anti-mouse-IR800 and a donkey anti-rabbit-IR700 (LICOR Biosciences, Lincoln, NE, USA). The picture corresponded to a fusion of those obtained with both dyes. The arrow in red located the a-tubulin, in green PRPF19 and in red the phosphorylated γ-H2AX. In (A) and (B), counts were done separately for both dyes. In all, these data indicated that i) the transfection affected the H2A.X-P/α-tubulin and the normalized α-tubulin amount (i.e. the cell survival and growth) since they were significantly different with those of control done with pBSK, ii) PB, PB.1–558, PB.NLS-1–558, Mm523, Mm409, Hs455 and Hs554 disp [file media-1.pdf]
